# Virulence of Two Isolates of *Meloidogyne enterolobii* (Guava Root-Knot Nematode) from North Carolina on Cotton Lines Resistant to Southern Root-Knot Nematode (*M. incognita*) and Reniform Nematode (*Rotylenchulus reniformis*)

**DOI:** 10.2478/jofnem-2023-0021

**Published:** 2023-06-23

**Authors:** Amanda G. Gaudin, Martin J. Wubben, Jack C. McCarty, Johnie N. Jenkins

**Affiliations:** USDA-ARS, Crop Science Research Lab, Genetics and Sustainable Agriculture Research Unit, 150 Twelve Lane, Mississippi State, MS 39762 USA.

**Keywords:** Cotton, resistance, root-knot nematode, Meloidogyne

## Abstract

*Meloidogyne enterolobii* [the guava root-knot nematode (RKN)] is an emerging plant-parasitic nematode that poses a threat to Upland cotton (*Gossypium hirsutum*) production in the southeastern United States. Like other RKN spp., *M. enterolobii* has a wide host range and proven ability to overcome resistance sources that have helped protect crops from other *Meloidogyne* spp., including the southern RKN (*Meloidogyne incognita*). In this study we evaluated the virulence of two North Carolina *M. enterolobii* isolates on Upland cotton germplasm lines having resistance quantitative trait loci (QTL) to RKN (M240 RNR, MRk-Rn-1) and/or reniform nematode (*Rotylenchulus reniformis*) (M713 Ren1, MRk-Rn-1) in comparison to their susceptible recurrent parents (DPL61, SG747). Multiple assays using eggs or J2 as inoculum demonstrated that both isolates reproduced equally well on all germplasm lines, producing reproductive factor (RF) values ≥ 6 on the otherwise nematode-resistant lines. Measurements of seedling growth in control and inoculated containers suggested that existing nematode-resistance QTL may offer a level of tolerance to *M. enterolobii* infection that should be further explored in greenhouse and field environments. *Meloidogyne enterolobii* infection of SG747 and MRk-Rn-1 showed nearly identical stages of symptom and nematode development over a time-course of 24 days. These data demonstrate that existing RKN and RN resistance QTL available in elite cotton varieties to producers are most likely insufficient in preventing yield loss due to *M. enterolobii* and that future research should focus on (i) understanding the *M. enterolobii*–cotton interaction at the molecular level, and (ii) screening novel germplasm collections to identify resistance loci.

The guava root-knot nematode (*Meloidogyne enterolobii*) is a sedentary endoparasitic species that parasitizes many important crops, including guava, tomato, sweetpotato, and cotton. Since its initial discovery in China, *M. enterolobii* has been identified in Africa, southeast Asia, and the Americas (Yang and Eisenback, 1983; Elling, 2013). Initially, the nematode was misidentified as two different species, *M. mayaguensis* and *M. enterolobii* with *M. mayaguensis* being documented as an important crop pest on tomato and peppers (Brito et al., 2004). However, it has since become accepted that *M. mayaguensis* and *M. enterolobii* are the same species of nematode based on the sequence identity of mitochondrial DNA (Xu et al., 2004; Castagnone-Sereno, 2012).

The *M. enterolobii* life cycle is like that of other *Meloidogyne* spp. (Elling, 2013). Mature females deposit eggs into a gelatinous matrix secreted by the rectal glands. Development within the egg results in the first-stage juvenile that molts inside the egg to become the infective second-stage juvenile (J2). The J2 infective life stage emerges from the eggs and infects host plant roots preferentially through the root tips and migrates intercellularly through the root cortex. The J2 becomes sedentary and a collection of feeding cells, called “giant cells,” are formed near the endodermis and function to supply nutrition. The giant cell is a single cell that undergoes multiple rounds of mitosis without cytokinesis, creating a large multinucleate cell specialized for nematode feeding. Molting through the J3 and J4 life stages results in mature female development and egg deposition. *M. enterolobii* reproduces by parthenogenesis and has a similar chromosomal makeup as the southern root-knot nematode (RKN) *M. incognita* (Cetintas et al., 2007; Collett et al., 2021). However, the time it takes to complete a full life cycle and reproduce is variable when compared to related species, sometimes taking longer and other times being significantly faster, depending on isolate genotype, soil temperature, host plant species, and other environmental factors (Collett et al. 2021).

In the contiguous United States, *M. enterolobii* was first reported in Florida, being initially classified as *M. mayaguensis*, where it was morphologically and molecularly differentiated from other common *Meloidogyne* spp., e.g., *M. incognita* and *M. arenaria* (Brito et al., 2004). In the late 2010s, *M. enterolobii* was discovered in North and South Carolina, and Louisiana (Ye et al., 2013; Rutter et al., 2019; Philbrick et al. 2020). Recently, *M. enterolobii* was intercepted in Louisiana on sweetpotato storage roots imported from North Carolina, confirming previous suspicions of a link between *M. enterolobii* spread and sweetpotato planting stock (Thiessen, 2018; Rezende et al., 2022). Because of its wide geographical distribution and host range, *M. enterolobii* is considered an important nematode species and is on the European and Mediterranean Plant Protection Organization (EPPO) quarantine list because of high pathogenicity (Castagnone-Sereno, 2012).

*M. enterolobii* is of special concern because it can infect and proliferate on crop varieties that have resistance to other RKN species. This ability has been confirmed in sweetpotato, tomato, potato, and soybean (Brito et al., 2007; Cetintas et al., 2008). In a study comparing *Meloidogyne* pathogenicity on tomato, *M. enterolobii* was found to cause more severe root galling than other *Meloidogyne* spp., including southern RKN (Cetintas et al., 2007). The *Mi-1* gene in tomato confers resistance to *M. incognita*, *M. arenaria*, and *M. javanica*; however, this gene was ineffective against *M. enterolobii* (Brito et al., 2007). Furthermore, the *N* gene in bell pepper and the *Tabasco* gene in pepper, both of which confer resistance to *M. incognita*, *M. arenaria,* and *M. javanica*, did not protect against *M. enterolobii* (Brito et al., 2007).

The potential spread of *M. enterolobii* is an emerging problem for cotton growers in the southeastern U.S. and management options need to be identified to prevent significant crop loss. Decades of research focused on developing cotton germplasm resistant to *M. incognita* and reniform nematode (RN) (summarized in McCarty et al., 2021) recently yielded the deployment of elite Upland cultivars resistant to both species. For *M. incognita*, resistance is mediated by two loci, *qMi-C11* and *qMi-C14*, whereas resistance to RN is largely controlled by a single locus on chromosome 21 (*Ren^barb2^*) (Gutiérrez et al., 2010; Wubben et al., 2017). With the availability of these nematode-resistant cultivars to cotton producers, it is vital that we understand how these resistance loci impact the infection and reproduction of *M. enterolobii* on cotton. Toward this end, our laboratory secured the necessary phytosanitary permits from the State of Mississippi Plant Advisory Board and the Animal and Plant Health Inspection Service (APHIS) to import two North Carolina isolates of *M. enterolobii* to test for potential differences in virulence on southern RKN and RN-resistant cotton germplasm lines.

## Materials and Methods

### Nematode cultures

Southern RKN (*Meloidogyne incognita*) race 3 and *M. enterolobii* cultures were maintained on the Upland cotton germplasm line “M8” in separate growth chambers. *Meloidogyne enterolobii* isolate “NC.1” was received from Dr. William Rutter (USDA-ARS) and isolate “18-5126” from Dr. Eric Davis (North Carolina State University) under APHIS permit number P526P-19-02617. Details describing the isolation and characterization of these isolates can be found in Rutter et al. (2021) and Schwarz et al. (2021), respectively. A quarantine permit was obtained from the State of Mississippi Department of Agriculture and Commerce that allowed experiments to be conducted in growth chambers at our facility. Soil and tissues of *M. enterolobii*-infected plants were autoclaved prior to disposal. Water used for washing soil from infected roots was passed through a #500-sieve before going down a drain.

### Cotton germplasm and inoculation experiments

The cotton germplasm lines M240-RNR (PI 592511) and M713 Ren1 (PI 665928) are highly resistant to RKN and RN, respectively (Shepherd et al., 1996; McCarty et al., 2013). The germplasm line M Rk-Rn-1 (PI 678938) combines the RKN and RN resistance present in M240-RNR and M713 Ren1 into a single genotype (McCarty et al., 2017). Susceptible checks were “Deltapine61” (DPL61; PI 607174) and “Sure-Grow747” (SG747; PI 656375). DPL61 served as the recurrent parent for M240-RNR, while SG747 was the recurrent parent for M713 Ren1 and M Rk-Rn-1.

*Meloidogyne* infection experiments were conducted using 16 oz plastic Solo® cups containing approximately 500 cm^3^ of an autoclaved mixture of 2-part sand:1-part Wickham sandy loam soil. Seeds were scarified, wrapped in wet paper towels, and incubated overnight at 30°C to promote germination. Seeds with radicles of around 0.5 cm were planted two per Solo® cup. For experiments using nematode eggs as inoculum, *M. incognita* or *M. enterolobii* eggs were collected from infected roots of culture cotton plants according to Hussey and Barker (1973). One day after planting, each Solo® cup was inoculated with 10,000 eggs delivered as 4 × 1 mL aliquots of a 2,500 egg/mL solution pipetted into four holes (two holes flanking each seedling). For experiments using *M. enterolobii* second-stage juveniles (J2) as inoculum, eggs of each isolate were collected as described above and layered over a #500 nylon mesh submerged in distilled water. Hatched J2 were collected daily over a period of 5 to 7 days. Each Solo® cup was inoculated with 2,000 J2 as 4 × 1 mL aliquots of a 500 J2/mL solution pipetted into four holes (two holes flanking each seedling). All experiments were performed in a Percival PGC-9/2 growth chamber at a constant temperature of 30°C under a 16 hr day/8 hr night cycle. Except for time course-dependent studies, all experiments were halted six weeks post-inoculation and root tissues collected. Gall severity was measured on a 1–10 scale (1=no galls present and 10=large galls present on entire root system). Root tissues were weighed and the eggs extracted, stained, and counted as previously described (Gaudin and Wubben, 2021). The reproductive factor (RF) was calculated as total eggs collected ÷ initial inoculum. Acid fuchsin staining and visualization of infected roots was performed as previously described (Wubben et al., 2020). Measurements of plant growth traits (stem height, shoot fresh weight, leaf number and node number) were performed six-weeks after inoculation. Stem height was measured from the stem base to the apical meristem.

### Statistical analyses

All experiments were conducted according to a completely randomized design with five to six replications per treatment. Data collected from each experiment were analyzed separately using the general linear model function and analysis of variance with SAS version 9.4 (SAS Institute). Egg per gram root values were log-transformed. Interaction effects were determined for nematode population × line for the initial three experiments, and timepoint × nematode × line was determined for the fourth experiment. Means were separated at *P* ≤ 0.05 using least squared means with the Tukey-Kramer adjustment for multiple comparisons.

## Results

### Reproduction of *Meloidogyne enterolobii* isolates on RKN and RN-resistant germplasm

**Figure 1:**
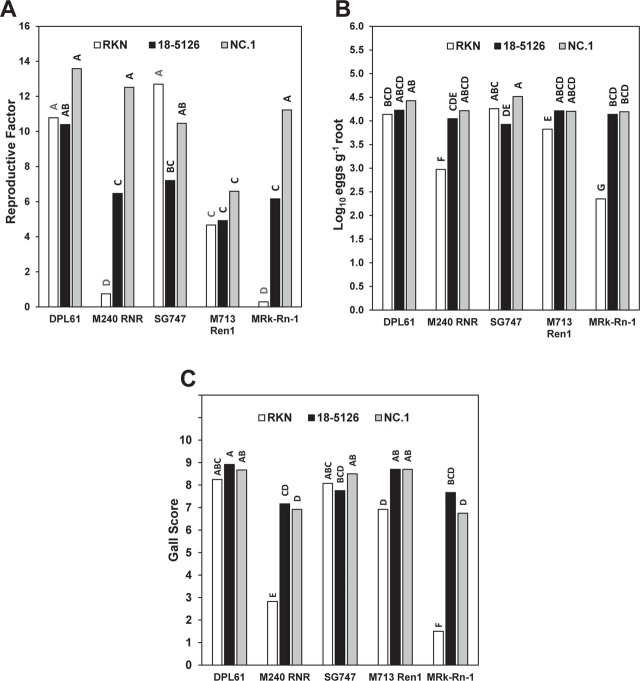
Virulence of *Meloidogyne enterolobii* isolates 18-5126 and NC.1 on cotton germplasm lines resistant to *M. incognita* (RKN) (M240 RNR), resistant to RN (M713 Ren1), or resistant to both RKN and RN (M Rk-Rn-1) in comparison to a greenhouse-maintained RKN race 3 population. Also included were nematode-susceptible obsolete cultivars Deltapine61 (DPL61) and SureGrow747 (SG747). Eggs were used as inoculum and virulence was measured as (A) RF, (B) log_10_ eggs g^−1^ root, and (C) gall score index (1–10 scale). Tukey-Kramer multiple comparison was used to determine significant differences between means at α = 0.05 as denoted by different letters (n = 6).

In our first assay, eggs were used as inoculum to compare the reproduction of *M. enterolobii* to that of RKN on susceptible (DPL61 and SG747) and RKN/RN resistant germplasm (M240 RNR, M713 Ren1, and M Rk-Rn-1). The interaction of cotton line with nematode isolate was significant at the α ≤ 0.05 for RF, log_10_(eggs g^−1^ root), and gall score (Fig. 1). As expected, the RF of *M. incognita* was significantly lower on M240 RNR and MRk-Rn-1 compared to the other germplasm lines (Fig. 1A). In contrast, both *M. enterolobii* isolates showed an RF > 6 on M240 RNR and MRk-Rn-1, with isolate NC.1 having a significantly greater RF versus isolate 18-5126 (Figure 1A). When inoculated with isolate NC.1, there was no difference in RF among DPL61, SG747, M240 RNR, and MRk-Rn-1 (Fig. 1A). Isolate 18-5126 showed similar RF values across all germplasm lines except for DPL61 which showed greater reproduction compared to the RKN/ RN-resistant lines but SG747 (Fig. 1A). Curiously, the RN-resistant line M713 Ren1 showed the lowest RF for isolate NC.1 versus all other lines (Fig. 1A).

The log_10_(eggs g^−1^ root) and gall score values showed trends similar to those of RF, with a few notable exceptions (Figs. 1A, B). M713 Ren1, in contrast to RF, showed no difference in log_10_(eggs g^−1^ root) or gall score between *M. enterolobii* isolates. Likewise, no differences between 18-5126 and NC.1 were observed for log_10_(eggs g^−1^ root) or gall score on M240 RNR and M Rk-Rn-1, in contrast to their respective RF values. In fact, the log_10_(eggs g^−1^ root) of *M. enterolobii*-inoculated M240 RNR, MRk-Rn-1, and M713 Ren1 were not different from RKN-inoculated DPL61 and SG747 (Fig. 1B). Overall, gall scores revealed a similar trend in which no differences were found in DPL61 and SG747 under the pressure of different nematode species (Fig. 1C). Lines M240 RNR and MRk-Rn-1 tended to show less severe galling in response to *M. enterolobii* compared to DPL61, SG747, and M713 Ren1; however, the galling observed was clearly within what would be expected in a susceptible genotype (Fig. 1C).

**Figure 2:**
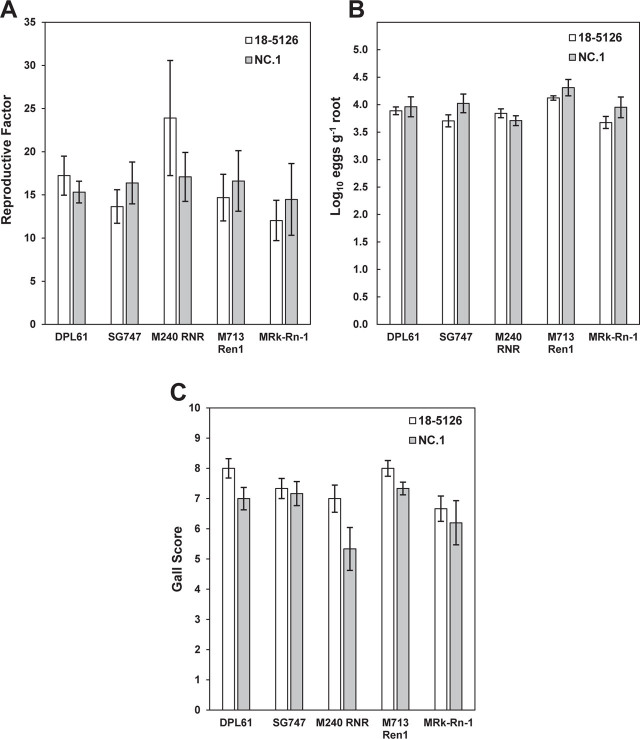
Virulence of *Meloidogyne enterolobii* isolates 18-5126 and NC.1 on cotton germplasm lines resistant to *M. incognita* (RKN) (M240 RNR), resistant to RN (M713 Ren1), or resistant to both RKN and RN (M Rk-Rn-1) using second-stage juveniles as inoculum. Also included were nematode-susceptible obsolete cultivars Deltapine61 (DPL61) and SureGrow747 (SG747). Virulence was measured as (A) RF ± SE, (B) log_10_ eggs g^−1^ root ± SE, and (C) gall score index ± SE (1–10 scale) of six replicates.

A second assay was performed using hatched J2 as inoculum to account for possible differences in egg hatch between isolates 18-5126 and NC.1. The results of this experiment showed that no significant differences in RF and log_10_(eggs g^−1^ root) were detected between isolates for any of the germplasm lines (Figs. 2A, B). The interaction effect of cotton line × *M. enterolobii* isolate was not significant; however, the isolates were significantly different for gall score, with 18-5126 generally producing a higher gall score than NC.1 (Fig. 2C). Based on the two assays, we concluded that isolates 18-5126 and NC.1 did not differ significantly in their virulence across the germplasm lines; therefore, only isolate NC.1 was used in subsequent experiments.

### Effects of *M. enterolobii* isolate NC.1 on cotton seedling growth

At the conclusion of both assays described above, we noticed that lines M240 RNR and MRk-Rn-1 tended to appear “healthier” compared to DPL61 and SG747 in terms of leaf number, stem height, and general appearance. To help determine the impact of *M. enterolobii* infection on cotton seedling growth, a third experiment was conducted that compared plant growth traits of inoculated plants to their uninoculated control counterparts. Because our experiments were limited to growth chambers, we decided to use the following measures: stem height (mm), shoot fresh weight (g), leaf number, and stem height/node number (Table 1).

Significant decreases in stem height due to *M. enterolobii* infection were observed for DPL61, SG747, and MRk-Rn-1 (Table 1). M240 RNR and M713 Ren1 also showed a decrease in stem height; however, this decrease was not significant. A similar pattern was observed for stem height/node number, where only M240 RNR and M713 Ren1 did not show a significant reduction in response to infection. Stem height/node number is commonly used as an indicator of stress on a cotton plant (Lu et al., 2014). Fresh shoot weight was only significantly impacted in SG747. For leaf number, *M. enterolobii* infection showed a significant effect for SG747 and MRk-Rn-1.

**Table 1.** Effect of *Meloidogyne enterolobii* infection on cotton growth under growth chamber conditions. Presented are mean ± SE after 6 weeks of growth under uninoculated (control) or inoculated conditions (n = 5).

		**Control**	**Inoculated**
**Stem Height (mm)**	DPL61	124.1 ± 3.2	79.3 ± 3.7^**^
	M240 RNR	147.8 ± 4.6	133.8 ± 22.8
	SG747	143.5 ± 2.8	89.6 ± 2.1^**^
	M713 Ren1	144.0 ± 17.0	131.0 ± 12.8
	MRk-Rn-1	157.6 ± 10.5	111.6 ± 5.4^**^
**Shoot Fresh Weight (g)**	DPL61	2.49 ± 0.13	1.94 ± 0.23
	M240 RNR	2.75 ± 0.23	2.01 ± 0.27
	SG747	3.23 ± 0.45	1.48 ± 0.19^**^
	M713 Ren1	2.58 ± 0.22	3.39 ± 0.42
	MRk-Rn-1	2.99 ± 0.74	1.83 ± 0.11
**Leaf Number**	DPL61	3.7 ± 0.1	3.8 ± 0.4
	M240 RNR	3.8 ± 0.1	3.5 ± 0.5
	SG747	3.5 ± 0.2	2.4 ± 0.3^**^
	M713 Ren1	3.9 ± 0.3	3.8 ± 0.3
	MRk-Rn-1	4.1 ± 0.3	3.1 ± 0.2^**^
**Stem Height/Node Number**	DPL61	28.9 ± 0.5	21.1 ± 1.8^**^
	M240 RNR	33.2 ± 1.9	33.2 ± 1.5
	SG747	40.0 ± 2.3	26.5 ± 0.9^**^
	M713 Ren1	31.7 ± 4.7	27.6 ± 2.2
	MRk-Rn-1	40.5 ± 2.3	29.5 ± 1.1^**^

** Significantly different (*P* ≤ 0.05) from corresponding control mean.

**Figure 3:**
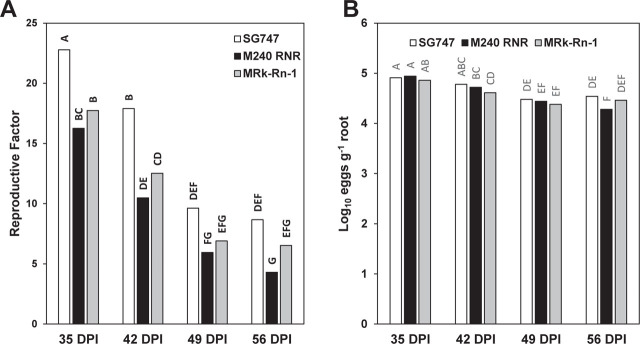
Time-point dependent reproduction of the *Meloidogyne enterolobii* isolate NC.1 on cotton germplasm lines M240 RNR and MRk-Rn-1 in comparison to the susceptible cultivar SureGrow747 (SG747). Total eggs were extracted at 35-, 42-, 49-, and 56-days post inoculation (DPI). Isolate NC.1 reproduction is presented as (A) RF (total eggs extracted/initial inoculum) and (B) log_10_ eggs g^−1^ root. Significant differences between means at α = 0.05 noted by different letters (n = 6).

Eggs were extracted from the inoculated plants in this experiment and were counted. The RF was similar between DPL61, SG747, and M240 RNR (25.1, 23.0, and 25.1, respectively). In contrast, M713 Ren1 and MRk-Rn-1 both showed RF values of 12.2, significantly lower than the others. However, this difference disappeared when log_10_(eggs g^−1^ root) was determined and showed no differences between any of the lines.

### Time-course reproduction of isolate NC.1

In a final assay, we measured *M. enterolobii* reproduction on SG747, M240 RNR, and MRk-Rn-1 at four time points: 35, 42, 49, and 56 days post-inoculation (DPI). Previous experiments had measured reproduction at six weeks (42-DPI); however, measuring at multiple time points may shed light on the relative speed of infection and development of *M. enterolobii* in different germplasm lines. In general, reproduction decreased over time for each germplasm line (Fig. 3). The first time point (35-DPI) showed the highest level of reproduction in each line and revealed a significantly higher RF in SG747 versus M240 RNR and MRk-Rn-1 (Fig. 3A). This difference was also observed at 42-DPI but largely disappeared by 49- and 56-DPI (Fig. 3A). Analysis of log_10_(eggs g^−1^ root) did not reveal a significant line × time point interaction, and only slight differences were detected between lines and time points (Fig. 3B). In contrast to the RF data, log_10_(eggs g^−1^ root) revealed no significant differences between lines within each time point, with the single exception of M240 RNR at 56-DPI.

### Time course of isolate NC.1 infection in SG747 and MRk-Rn-1 seedlings

**Figure 4:**
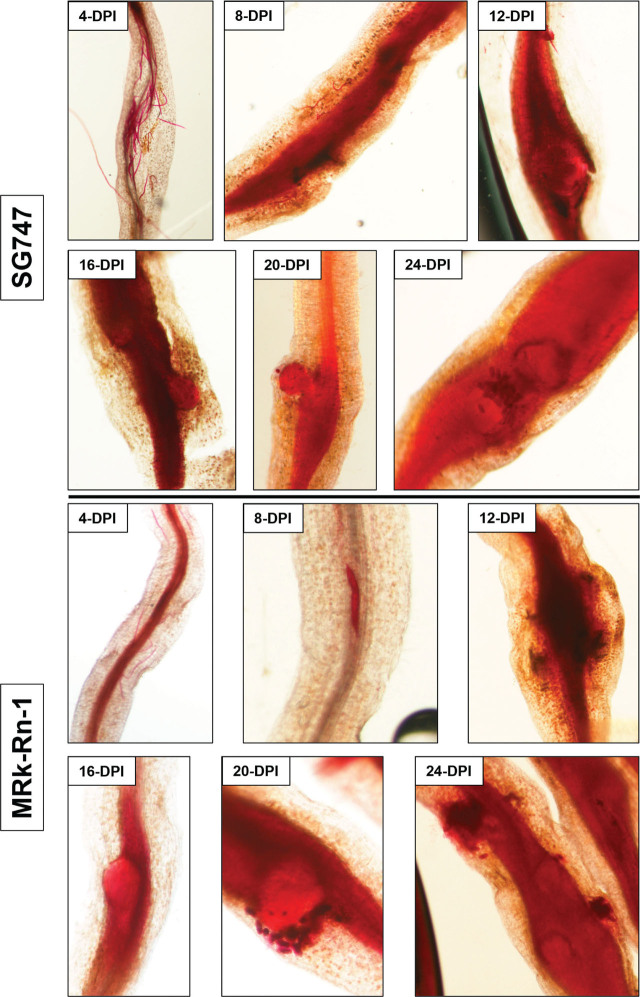
Time-course of *Meloidogyne enterolobii* isolate NC.1 infection of SureGrow747 and MRk-Rn-1. Infected roots were collected at 4-, 8-, 12-, 16-, 20-, and 24-days after inoculation (DAI) and stained with acid fuchsin.

*Meloidogyne enterolobii* isolate NC.1-infected SG747 and MRk-Rn-1 roots were collected and stained with acid fuchsin at 4, 8, 12, 16, 20, and 24 DPI (Fig. 4). The timing of infection and rate of juvenile development was nearly identical between SG747 and MRk-Rn-1. At 4-DPI, infective J2 had penetrated the root tips of both lines and migrated intercellularly through the cortex. By 8-DPI, root swelling was evident and sedentary parasitic J2 were observed. Nematode development progressed rapidly after 8-DPI such that severe root galling and J4 females were present in both lines at 12- and 16-DPI. Mature females and egg deposition was evident by 20-DPI and manifested throughout the root system of both lines by 24-DPI.

## Discussion

*Meloidogyne enterolobii* is an emerging pathogen in the cotton production areas of the southeastern U.S. Reports from Brazil indicated that cotton varieties expressing resistance to southern RKN were susceptible to populations of *M. enterolobii* (Galbieri et al., 2020). Multiple isolates of *M. enterolobii* have been collected from the Carolinas and evaluated in greenhouse experiments for differences in virulence on a range of sweetpotato genotypes (Schwarz et al., 2020; Rutter et al., 2021). In this study, we evaluated two such isolates regarding their reproduction on cotton germplasm lines having strong resistance to southern RKN, mediated by *qMi-C11* and *qMi-C14*, and/or RN, mediated by the *Ren^barb2^* QTL.

In general, the two North Carolina isolates used in our study, “18-5126” and “NC.1,” reproduced to similar levels on all cotton germplasm lines tested, with slight variations between experiments. Both isolates were able to overcome southern RKN and RN resistance QTL and induce severe root galling and high RF values. These data suggest the 18-5126 and NC.1 isolates are genetically similar and may originate from the same original introduction into North Carolina. A similar conclusion was determined by testing four North Carolina isolates on sweetpotatoes, which showed no differences in virulence (Schwarz et al., 2020). In contrast, a South Carolina *M. enterolobii* isolate, “SC.1,” was discovered that showed zero reproduction on Upland cotton (Rutter et al., 2021); however, the nature of this cotton resistance to SC.1 remains unknown. *Meloidogyne enterolobii* has a history of evading or overcoming RKN spp. resistance in many crops, making the results of this study not wholly unexpected. In a study on sweetpotatoes, *M. enterolobii* reproduced well on multiple cultivars that were previously identified as moderately resistant to RKN (Brito et al., 2020). The tomato *Mi-1* resistance gene is effective for the management of southern RKN and two other root-knot species, *M. arenaria* and *M. javanica*. Despite this multi-specie protection, *M. enterolobii* reproduced on *Mi-1* tomato cultivars like susceptible tomato varieties (Kiewnick et al., 2009). Similarly, the *N* gene in peppers provides resistance to southern RKN, *M. arenaria*, and *M. javanica*, but this gene was also overcome by *M. enterolobii* (Kiewnick et al., 2009).

We determined existing cotton nematode resistance genes were ineffective against *M. enterolobii*, much like the *Mi-1* gene in tomato and the *N* gene in pepper. The ability of *M. enterolobii* to overcome the resistance of related nematode species, particularly *M. incognita*, is problematic for future nematode management efforts (Kiewnick et al., 2009). In regard to cotton, the mechanism of defense evasion needs to be examined to better determine potential control options. For example, on the molecular level, *M. enterolobii* is either going undetected by qMi-C11 and qMi-C14, or these QTL are being triggered, but the resulting defense is ineffective against *M. enterolobii*, possibly via effector triggering susceptibility. Understanding how *M. enterolobii* is overcoming the resistance is important for continuing the search for novel resistance.

With the knowledge that current Upland cotton RKN and RN-resistant genes are ineffective, the search for *M. enterolobii* resistance will require looking elsewhere and searching for novel genes and allele combinations. Extensive screening has been a successful, albeit labor-intensive, tool used to identify nematode resistance genes in cotton. For example, the currently used RN resistance was originally identified in *G. barbadense* accession GB-713 during such germplasm screenings, leading to the development of RN-resistant Upland cotton lines (Robinson et al., 2004). Common cotton breeding methods are effective for novel cotton line development. In early RKN resistance screenings, two moderately resistant lines (Clevewilt-6 and Mexico Wild Jack Jones) were crossed to develop the highly resistant line Auburn 634, which had *qMi-C11* and *qMi-C14* resistance loci (Shepherd, 1974; Gutiérrez et al., 2010). Other tools like random mating are often used to break negative genetic linkages by outcrossing between multiple diverse parents (Gutiérrez et al., 2006). Linkages are broken by forming new combinations of alleles and potentially new phenotypes. For example, these novel combinations can then be screened for potential resistance or tolerance to *M. enterolobii*. Historically, superior gene combinations have been generated in randomly mated populations (Gutiérrez et al., 2006).

In summary, the present study indicated that existing nematode resistance QTL in cotton are ineffective against *M. enterolobii*. Due to experimental restrictions limiting work to only growth chambers, these data cannot address questions regarding the effect of season-long exposure of cotton germplasm lines to *M. enterolobii* infection. In addition to germplasm screening for resistance, future work should incorporate commercially available cotton varieties having the same nematode resistance QTL as described in this study, into field trials to examine any potential tolerance afforded by existing resistance QTL.
